# Pharmacist Computer Skills and Needs Assessment Survey

**DOI:** 10.2196/jmir.6.1.e11

**Published:** 2004-03-29

**Authors:** Robert M Balen, Peter J Jewesson

**Affiliations:** ^1^Pharmaceutical Sciences Clinical Service UnitVancouver General HospitalCanada; ^2^Pharmaceutical Sciences Clinical Service UnitVancouver General HospitalUniversity of British ColumbiaCanada

**Keywords:** Computer literacy, pharmacy, clinical informatics, needs assessment, pharmacists, survey

## Abstract

**Background:**

To use technology effectively for the advancement of patient care, pharmacists must possess a variety of computer skills. We recently introduced a novel applied informatics program in this Canadian hospital clinical service unit to enhance the informatics skills of our members.

**Objective:**

This study was conducted to gain a better understanding of the baseline computer skills and needs of our hospital pharmacists immediately prior to the implementation of an applied informatics program.

**Methods:**

In May 2001, an 84-question written survey was distributed by mail to 106 practicing hospital pharmacists in our multi-site, 1500-bed, acute-adult-tertiary care Canadian teaching hospital in Vancouver, British Columbia.

**Results:**

Fifty-eight surveys (55% of total) were returned within the two-week study period. The survey responses reflected the opinions of licensed BSc and PharmD hospital pharmacists with a broad range of pharmacy practice experience. Most respondents had home access to personal computers, and regularly used computers in the work environment for drug distribution, information management, and communication purposes. Few respondents reported experience with handheld computers. Software use experience varied according to application. Although patient-care information software and e-mail were commonly used, experience with spreadsheet, statistical, and presentation software was negligible. The respondents were familiar with Internet search engines, and these were reported to be the most common method of seeking clinical information online. Although many respondents rated themselves as being generally computer literate and not particularly anxious about using computers, the majority believed they required more training to reach their desired level of computer literacy. Lack of familiarity with computer-related terms was prevalent. Self-reported basic computer skill was typically at a moderate level, and varied depending on the task. Specifically, respondents rated their ability to manipulate files, use software help features, and install software as low, but rated their ability to access and navigate the Internet as high. Respondents were generally aware of what online resources were available to them and Clinical Pharmacology was the most commonly employed reference. In terms of anticipated needs, most pharmacists believed they needed to upgrade their computer skills. Medical database and Internet searching skills were identified as those in greatest need of improvement.

**Conclusions:**

Most pharmacists believed they needed to upgrade their computer skills. Medical database and Internet searching skills were identified as those in greatest need of improvement for the purposes of improving practice effectiveness.

## Introduction

Pharmacy is an information intensive profession. The availability of affordable computers and the advancement of information technology have resulted in our ability to rapidly and effectively access, retrieve, analyze, share, and store large volumes of information pertinent to patient care [1].

To use technology effectively for the advancement of patient care, pharmacists must possess a variety of computer skills. We recently introduced a novel program in our clinical service unit at this hospital, aimed at the improvement of the applied informatics abilities of our members. In keeping with the broad mandate to introduce this program, this study was conducted to gain a better understanding of the computer skills and needs of our pharmacists.

## Methods

This study involved a survey of all practicing licensed pharmacists at Vancouver Hospital and Health Sciences Centre, a multi-site, 1500-bed, acute-adult-tertiary care Canadian teaching hospital in Vancouver, British Columbia. This survey was conducted in May 2001.

### Subjects

The participants of this study were licensed BSc and PharmD pharmacists at two of our hospital sites (Vancouver General Hospital, University of British Columbia Hospital). A computerized drug distribution system has been in use at both hospitals for at least 10 years.

### Survey Instrument

To assess the computer skill sets of the participants, an 84-question written survey was created (Appendix 1). A review of the literature was undertaken to identify previously published surveys [2,4- 8]. None of these published instruments met all our needs. Some knowledge and experiential domains were adopted from this previous literature, and questions that were applicable to our purposes were either adopted or modified for inclusion in our survey. The majority of questions were developed internally by author consensus. Our survey was constructed to elicit information in nine primary domains relevant to identifying training needs and system barriers to the expanded use of technology in clinical practice. These domains were: 1) computer experience; 2) computer anxiety; 3) computer vocabulary; 4) basic computer skills; 5) communications; 6) Internet skills; 7) clinical database information retrieval; 8) access to computers; 9) anticipated future needs. Five-point scales were employed where self-assessment questions were posed. Clinical database and search-engine questions were limited to the hardware, software, and online resources that were currently available to our members.

The survey was distributed to the mailboxes of 106 casual, part-time, and full-time staff pharmacists at the two hospital sites. A covering letter was attached explaining the rationale for the survey and the anonymity of results, and requesting return of the survey within two weeks.

### Data Analysis

Survey data were entered into a relational database (SPSS 10.1) [3] for the purposes of analysis. Incomplete surveys were included in the analysis and proportional data were expressed in terms of the number of respondents who answered a particular question. Descriptive analysis was also undertaken with the support of this software.

## Results

Of the 106 surveys distributed to the pharmacists, 58 surveys (55% of total) were returned within the two-week study period.

### Demographics

Surveys were completed by 58 pharmacists, including 20 (35%) residency-trained BSc (Pharm) pharmacists, 18 (31%) non-residency-trained pharmacists, 9 (16%) advanced degree (PharmD) pharmacists, 6 (10%) pharmacy supervisors, and 2 (3%) others. Pharmacist classification was undeclared for 3 (5%) respondents.

Twenty-six (45%) respondents had been in pharmacy practice for a period of 5 years or less. Eighteen (31%) respondents had been in practice for 6 to15 years, and 12 (21%) had been in practice for more than 15 years. Two (3%) respondents did not state duration of practice.

### Computer Experience

#### General

Forty-five (79%) of 57 respondents had received no formal computer training. Fifty-two respondents (93%) owned a home computer. Thirty (52%) respondents used their home computers as much as 5 hours per week, and 23 (40%) respondents used their computers 6 to 15 hours per week. Only 12 (21%) respondents had completed some formal computer training.

Of those who provided a response, 27 (47%) respondents used work computers as much as 5 hours per week for activities other than drug distribution purposes, and a remaining 28 (48%) respondents used work computers 6 to15 hours per week for activities other than drug distribution purposes.

Thirty-nine (67%) respondents had never used a handheld computer at work or at home (e.g. a Palm-based device), but 16 (28%) used this type of device daily.

#### Software

Pharmacists were asked to state how frequently they used six general types of software packages. Of those who provided a response, 47 (81%) used e-mail software at least once daily, and 20 (35%) used word processing software daily. The vast majority (>90%) of respondents did not use any statistical analysis or presentation software.

Forty-two (72%) respondents used the patient-care information computer for prescription processing on a daily basis. Forty-nine (85%) respondents used this computer system to review laboratory test results on a daily basis.

**Figure 1:**
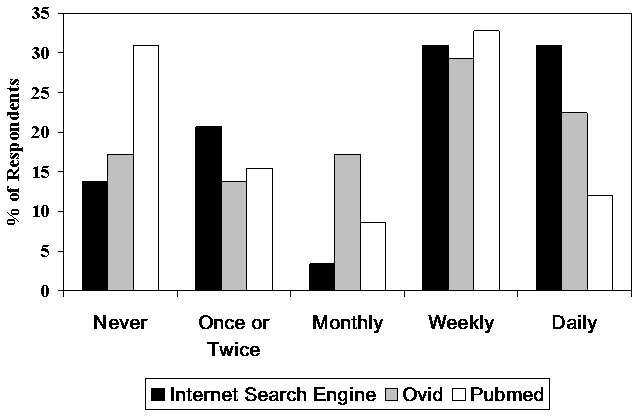
Frequency of search engine use by respondents (N= 58)

Figure 1 shows the frequency of use, according to respondent, of three types of search engines (i.e. an Internet search engine such as Google, or the specific medical database search engines Ovid or PubMed) for the purpose of finding clinical information. Thirty-nine (67%) respondents used one or more of the search engines at least on a weekly basis. A general Internet search engine such as Google was the most commonly used vehicle for finding clinical information according to 36 of 58 respondents (62%), followed by Ovid (30, 51%), and PubMed (26, 45%).

### Computer Literacy and Anxiety

**Figure 2:**
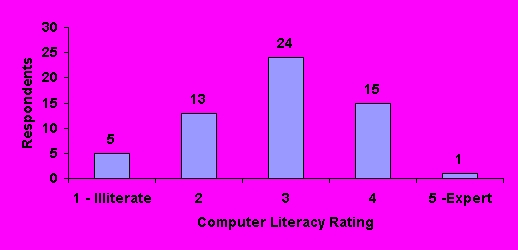
Computer literacy rating by respondents (N=58)

**Figure 3:**
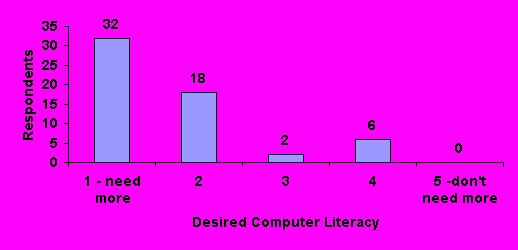
Desired computer literacy rating by respondents (N=58)

Using 5-point scales, pharmacists were asked to rate their current computer literacy and to compare this to their desired level of literacy (Figure 2, Figure 3). Respondents most commonly (24, 41%) ranked themselves as a "3" on the 5-point computer literacy scale. The majority (32, 55%) felt they needed more training to improve their computer literacy.

**Figure 4:**
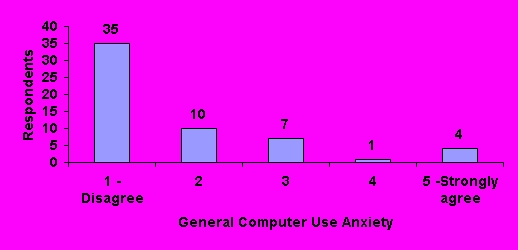
General computer use anxiety rating by respondents (N=57)

Pharmacists were asked whether or not the use of computers for purposes other than prescription processing or reviewing patient care information was anxiety provoking. Thirty-five (61%) respondents stated it was not; the remaining respondents stated that computer use caused varying degrees of anxiety (Figure 4).

### Computer Vocabulary

Pharmacists were asked to indicate their ability to define ten computer- and software-related terms. Although there appeared to be an understanding of most terms, 35 (61%) respondents were unable to describe a local area network (LAN), 26 (46%) were unfamiliar with PDF documents, 25 (44%) could not describe network drives, and 23 (40%) were unfamiliar with the term "URL address."

### Basic Computer Skills

Using a 5-point scale, pharmacists were asked to rate their ability to perform a variety of basic computer skills. The majority of respondents (42, 74%) rated their overall file management skills as "3" or greater. Twenty (35%) respondents stated that the specific computer task posing the greatest difficulty was file manipulation (e.g. copying and pasting a file or folder), 21 (37%) stated it was using software help features, and 25 (44%) stated it was installing software.

### Communications

At the time of this survey, all pharmacists at Vancouver Hospital and Health Sciences Centre had active hospital-based e-mail accounts. All respondents stated they had the ability to receive and read e-mail with varying degrees of skill, although 4 (7%) respondents were unable to send e-mail. Other reported difficulties related to e-mail were sending attachments, 11 (19%) unable; setting up a group mailing list, 13 (23%) unable; making mailboxes for saving and organizing important e-mail messages, 19 (33%) unable; keeping copies of sent e-mail, 10 (18%) unable; and automatically sorting e-mail with filtering rules, 21 (37%) unable.

### Internet Skills

Using a 5-point scale, pharmacists were asked to rate their ability to perform three basic Internet skills. The majority (46, 81%) of respondents rated themselves as "4" or greater in terms of their ability to access Web sites by typing the URL. Thirty-five (60%) rated themselves as "4" or greater in terms of ability to maintain a list of Web sites using the Web browser bookmark feature, and 12 (21%) rated themselves as a "1" (no ability). Thirty-one (54%) respondents rated themselves as "4" or greater in terms of ability to download files from online sources.

### Clinical Database Information Retrieval

Skill in using search engines varied widely among the pharmacists. Eleven (19%) of those who responded were unable to use the OVID search engine, and 15 (26%) were unable to use the PubMed search engine. Fourteen (24%) respondents had expert-level ability with the OVID search engine, and 7 (12%) had expert-level ability with the PubMed search engine. The remaining respondents had varying degrees of ability with each of these search engines. Twenty-three (40%) survey participants could not explain the differences between the OVID and PubMed search engines.

### Online Resources Awareness and Ability

**Figure 5:**
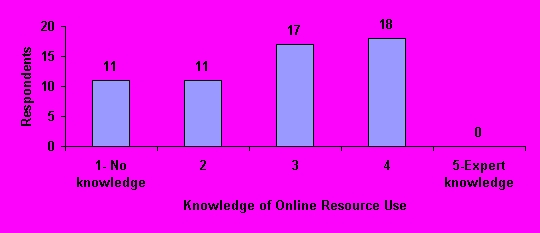
Knowledge of online resource rating by respondents (N=57)

The majority of respondents had some awareness of what online resources were available to them at our hospital for drug-related problems encountered in practice. On a 5-point scale, 43 pharmacists (75% of respondents) rated their awareness of resources as "3" or greater. Although no respondents rated themselves as having expert (i.e. "5") knowledge regarding use of online resources, 35 (61%) rated themselves as "3" or greater (Figure 5). Eleven (19%) respondents reported that they had no knowledge of what online resources to use for various drug-related problems.

On a 5-point scale, 27 (46%) respondents rated their frequency of use of Clinical Pharmacology as "4" or greater. Fifteen (26%) respondents used this reference frequently, e.g. weekly, and 9 (16%) never used this reference. When respondents were asked to rate their ability to use this reference, the most common response was "4" (19, 33%). Eighteen (31%) respondents rated their ability to use this reference as "poor," and only 2 (3%) rated themselves as expert.

On a 5-point scale, 18 (31%) respondents rated their frequency of use of MD Consult as "4" or greater. Seven (12%) used this reference frequently, e.g. weekly, and 14 (24%) never used this reference. When respondents were asked to rate their ability to use this reference, the most common response was "poor" (21, 36%). Fifteen (26%) respondents rated their ability to use this reference as "4," and only 2 (3%) rated themselves as expert.

On a 5-point scale, only 8 (14%) respondents rated their frequency of use of UpToDate as a "4" or greater. Six (10%) respondents used this reference frequently, e.g. weekly, and 28 (48%) never used this reference. When respondents were asked to rate their ability to use this reference, the most common response was "poor" (29, 50%). Twenty-three (40%) respondents rated their ability to use this reference as "2" or "3," and only 2 (3%) rated themselves as expert.

### Anticipated Future Needs

Using a 5-point scale (1 = no need; 5 = significant need), pharmacists were asked to rate their anticipated needs for nine independent computer skills (Appendix 1). In descending rank order, respondents rated their needs as "significant" for medical database search, 31, (53%); Internet search 30, (52%); Web browser navigation 25, (43%); advanced e-mail management 21, (36%); presentation software 20, (35%); word processing 17, (29%); database software 13, (22%); statistical software 10, (17%); and spreadsheet software 10, (17%).

**Figure 6:**
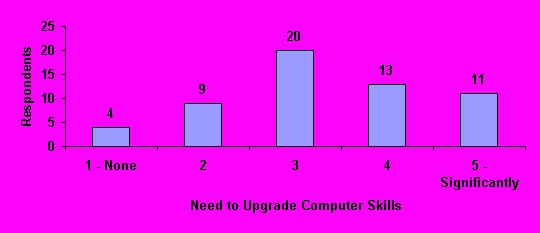
Need to upgrade computer skills to perform job more effectively (N=57)

When asked to identify their need for general computer skill upgrading to more effectively perform their jobs, 44 (77%) respondents rated their need as "3" or greater, and 11 (19%) rated their need as "significant" (Figure 6).

## Discussion

In addition to having strong clinical skills, pharmacists must be able to use resources effectively if they are to provide optimal patient care [10]. With the introduction of computers and access to the Internet, pharmacists must also possess the necessary computer skills to efficiently manage the high volume of information now available to them. Unfortunately, a standard definition of computer literacy and valid dimensions of computer competency for pharmacy practice have yet to be delineated.

In this study, we assessed the self-reported capabilities and needs of our pharmacists in an effort to optimize our staff training and resource development strategies. To our knowledge, this is the first published report to characterize the self-reported computer skills and needs of hospital pharmacists.

Our survey responses reflect the opinions of hospital pharmacists with a broad range of education and pharmacy practice experience. We found that most respondents had home access to personal computers and regularly used computers in the work environment for drug distribution, information management, and communication purposes. Few pharmacists reported experience with handheld computers. Software use experience varied according to application. Although patient-care information software and e-mail were commonly used, experience with spreadsheet, statistical, and presentation software was negligible. The pharmacists were familiar with Internet search engines, and their use was reported to be the most common method of seeking clinical information online. Although many pharmacists rated themselves as being generally computer literate and not particularly anxious about using computers, the majority believed that they required more training to reach their desired level of computer literacy. Lack of familiarity with computer-related terms was prevalent. Self-reported basic computer skill was typically of moderate level, and varied depending on the task. Specific file management tasks were commonly described as difficult, but most respondents had little difficulty with Internet access and navigation. Respondents were generally aware of what online resources were available to them, and Clinical Pharmacology was the most commonly employed reference. In terms of anticipated needs, most pharmacists believed they needed to upgrade their computer skills. Although respondents rated their skills in medical database and Internet searching as high, these skills were identified as in greatest need of further improvement for the purposes of improving practice effectiveness.

Although it is desirable for pharmacist practitioners to graduate with a minimum set of computer and information management skills, informatics remains an uncommon component of most pharmacy or medical school curricula [11,12]. Poikonen recently reported that of 86 US PharmD programs surveyed, the use of computers to assist in treatment decision-making occurred in less than half the schools that responded. Only 13% of schools employed an informatics faculty member [11]. The majority of our pharmacist members graduated from our local university (University of British Columbia) where informatics is not a formal component of the core undergraduate or graduate programs. In addition, the majority of respondents stated they had received no formal computer training. This probably affected the level of computer skills reported by this cohort.

Our study suffers from some methodological limitations. The anonymous survey involved a self-reporting of computer skills and needs; thus, the results must be considered subjective only. We sought the general impressions of our pharmacists, and utilized a simple rating scale in an attempt to quantify the respondents' perceptions of their abilities. We did not define all options between extreme or absolute scale points on the questionnaire, and this may have produced a tendency for respondents to select intermediary scores. No additional objective measurements (e.g. tests, observation of actual computer activities) were undertaken, nor was a validated survey available for use. Accordingly, we cannot directly compare our results with those of any other group. Although we achieved a relatively good response rate, the survey nevertheless involved a small non-randomized local sample of hospital pharmacists from two sites of a single health-care organization. Therefore, extrapolation of results to other practice environments must be done with caution.

In summary, this study has provided us with valuable insight into the current status of the computer skills and needs of our pharmacists. We will utilize this information to address the applied informatics needs of our members and help them use technology to enhance their knowledge, manage patient-care information, and improve their practice effectiveness. We recommend other groups consider undertaking a similar assessment of computer skills and needs, particularly if considering the implementation of an applied informatics program.

## Multimedia Appendix 1

Survey Form

## Abbreviations

CSU: Clinical services unit
PCIS: Patient care information system
VHHSC: Vancouver Hospital and Health Sciences Centre
