# Supplementary material for: Pharmacist Computer Skills and Needs Assessment Survey
Source: J Med Internet Res. 2004 Mar 29;6(1):e11. doi: 10.2196/jmir.6.1.e11 (PMC1550586; doi:10.2196/jmir.6.1.e11)
Supplement: Supplementary file 1 [file jmir_v6i1e11_app1.pdf]

Appendix 1. Survey form

VHHSC CSU Pharm. Sci. Computer User Survey - Please circle the most appropriate response to each of the following

This is an <u>anonymous</u> survey. The intent is to gain a better understanding of the computer skill sets of the deptartment members and to use this information to determine our training and resource development strategies. Dr. Robert Balen will be collating results which will be presented to department members at regular meetings. Please be honest with your replies. We recognize that computer skills vary between individuals.

Demographics

How many years have you been in pharmacy practice?	0-5	6-10	11-15	>15	
What is your current classification?	Pharm 1	Pharm II	Pharm III	Pharm IV	Other
Do you have a computer at home?	Yes	No			
If so, what operating system do you use at home?	Win 95	Win 98 SE	Win 2000/NT	Linux	Mac
Have you had any formal computer training (ie courses of some sort)?	Yes	No			
If so please indicate which courses you have taken:					
How many hours a week do you use a computer in the following settings:					
At work (excluding PCIS order entry)	0 h	> 0 - 5 h	6 - 10 h	11-15 h	> 15 h
Home	0 h	> 0 - 5 h	6 - 10 h	11-15 h	> 15 h

Computer Experience

Please respond to each of the following statements:

I have used a word processor (e.g. MS Word) to compose a text document	1 never	2 once or twice	3 monthly	4 weekly	5 daily
I have used a presentation program (e.g. Powerpoint) to create a presentation	1 never	2 once or twice	3 monthly	4 weekly	5 daily
I have used a spreadsheet program (e.g. Excell)	1 never	2 once or twice	3 monthly	4 weekly	5 daily
I have used a database program (e.g. Access)	1 never	2 once or twice	3 monthly	4 weekly	5 daily
I have used a statistical analysis program (e.g. SPSS)	1 never	2 once or twice	3 monthly	4 weekly	5 daily
I use an email program (e.g. GroupWise)	never	monthly	weekly	daily	multiple times daily
I have used the hospital computer to process a doctors order	1 never	2 once or twice	3 monthly	4 weekly	5 daily
I have used the hospital computer to look up laboratory test results	1 never	2 once or twice	3 monthly	4 weekly	5 daily
I have used other special features on the hospital computer system e.g. hotlists	1 never	2 once or twice	3 monthly	4 weekly	5 daily
I have used an internet search engine (e.g. Google) to find clinical information	1 never	2 once or twice	3 monthly	4 weekly	5 daily

I have searched a medical database using the OVID search engine	1 never	2 once or twice	3 monthly	4 weekly	5 daily
I have searched a medical database using the PubMed search engine	1 never	2 once or twice	3 monthly	4 weekly	5 daily
I have used a handheld computer (e.g. Palm) at work or at home	1 never	2 once or twice	3 monthly	4 weekly	5 daily

Computer Anxiety

How would you rate your overall computer literacy?	1 illiterate	2	3	4	5 expert
How does your current level of computer literacy compare to your desired level of computer literacy?	1 need more	2	3	4	5 don't need more
Are you anxious about the use of computers for purposes other than PCIS?	1 disagree	2	3	4	5 strongly agree

Computer Vocabulary

Do you feel you are able to describe the following terminology?

Hard drive	No	Yes
Network drive	No	Yes
RAM	No	Yes
Local area network (LAN)	No	Yes
Workstation	No	Yes
Computer virus	No	Yes
Multi-tasking	No	Yes
URL Address	No	Yes
Hardware vs. software	No	Yes
PDF file	No	Yes

Basic Computer Skills

Please rate your ability to do each of the following tasks:

Create a file or a folder	1 unable	2	3	4	5 expert
Rename a file or a folder	1 unable	2	3	4	5 expert
Move a file or folder	1 unable	2	3	4	5 expert
Copy a file or folder	1 unable	2	3	4	5 expert
Copy and paste a file or folder	1 unable	2	3	4	5 expert
Install a software program	1 unable	2	3	4	5 expert
Find answers using on-line help features	1 unable	2	3	4	5 expert
Switch between open applications	1 unable	2	3	4	5 expert

Search for files and directories on a computer	1 unable	2	3	4	5 expert
Minimize and maximize windows	1 unable	2	3	4	5 expert
Overall, I would rate my file management skills as:	1 unable	2	3	4	5 expert

Communications

Please rate your ability to do each of the following tasks:

Receive and read email	1 unable	2	3	4	5 expert
Send email	1 unable	2	3	4	5 expert
Send a document as an email attachment	1 unable	2	3	4	5 expert
Set-up a group mailing list	1 unable	2	3	4	5 expert
View an email attachment that I receive from others	1 unable	2	3	4	5 expert
Save an email attachment sent to me by someone	1 unable	2	3	4	5 expert
Make mailboxes for saving and organizing important email messages	1 unable	2	3	4	5 expert
Manage email messages by creating folders and filtering rules	1 unable	2	3	4	5 expert
Keep copies of email messages that I send to others	1 unable	2	3	4	5 expert

Internet Skills

Please rate your ability to do each of the following tasks:

Open a web page by typing in an web address	1 none	2	3	4	5 expert
Download files from an internet website	1 none	2	3	4	5 expert
Keep track of web sites using bookmarks	1 none	2	3	4	5 expert

Clinical Database Information Retrieval

Please rate your ability to do each of the following tasks:

Use the OVID search engine	1 unable	2	3	4	5 expert
Use the PubMed search engine	1 unable	2	3	4	5 expert
Explain the difference between OVID and PubMed search engines	1 unable	2	3	4	5 expert
Do you know which on-line resources are available to you at VHHSC?	1 no	2	3	4	5 know them all
Do you know what online resource to use for each type of drug problem you may encounter?	1 no	2	3	4	5 I'm an expert

Do you use **Clinical Pharmacology 2000**?	1 never	2	3	4	5 frequently e.g weekly
Are you familiar with what kind of information this database provides?	1 no	2	3	4	5 expert knowledge
How would you rate your ability to use this database?	1 poor	2	3	4	5 expert
Do you use **MD Consult**?	1 never	2	3	4	5 frequently e.g weekly
Are you familiar with what kind of information this database provides?	1 no	2	3	4	5 expert knowledge
How would you rate your ability to use this database?	1 poor	2	3	4	5 expert
Do you use **UpToDate**?	1 never	2	3	4	5 frequently e.g weekly
Are you familiar with what kind of information this database provides?	1 no	2	3	4	5 expert knowledge
How would you rate your ability to use this database?	1 poor	2	3	4	5 expert

Access to computers

How difficult is it for you to get access to a computer at work?	1 impossible	2	3	4	5 easy

Rank Your Anticipated Future Need

Please rate your anticipated future needs for the following skills according to their relevance to your professional job effectiveness:

Word processing software skills	1 no need	2	3	4	5 significant need
Presentation software skills	1 no need	2	3	4	5 significant need
Spreadsheet software skills	1 no need	2	3	4	5 significant need
Database software skills	1 no need	2	3	4	5 significant need
Web browser navigation skills	1 no need	2	3	4	5 significant need
Internet search skills	1 no need	2	3	4	5 significant need
Medical database search skills	1 no need	2	3	4	5 significant need
Statistical analysis software skills	1 no need	2	3	4	5 significant need

Advanced e-mail management skills	1 no need	2	3	4	5 significant need

How would you rate the following statement

How much do you feel you need to upgrade your computer skills in order to perform your job more effectively?	1 none	2	3	4	5 significantly

What is the biggest technology-related challenge that you face at work?
